# Treatment of obstructive jaundice induced by non-hodgkin lymphoma with EUS-guided transgastric anterograde common bile duct stenting: Technical case report and literature review

**DOI:** 10.3389/fsurg.2022.1031718

**Published:** 2023-01-06

**Authors:** Lingzhi Yuan, Xiao Shi, Hongbo Shan, Dinghua Xiao, Xiaoyan Wang, Fen Wang

**Affiliations:** ^1^ Department of Gastroenterology, The Third Xiangya Hospital, Central South University, Changsha, China; ^2^ Hunan Key Laboratory of Non-Resolving Inflammation and Cancer, Central South University, Changsha, China; ^3^ Department of Endoscopy, Sun Yat-Sen University Cancer Hospital, Guangzhou, China

**Keywords:** endoscopic ultrasound, biliary stent, lymphoma, obstructive jaundice, treatment

## Abstract

**Background:**

Non-Hodgkin lymphoma (NHL) is a rare cause of biliary obstruction. The optimum treatment for these patients is unclear. Lymphoma-associated obstructive jaundice is generally managed with open surgery, Endoscopic retrograde cholangiopancreatography (ERCP), or Percutaneous transhepatic biliary drainage. Here, we present the first description of EUS-guided anterograde common bile duct stenting *via* the stomach for obstructive jaundice associated with NHL.

**Patient and methods:**

A 58-year-old male patient who had been undergoing chemotherapy for NHL was admitted to our institution for severe obstructive jaundice. The patient's hepatic function indicators were: alanine aminotransferase 211 U/L, aspartate aminotransferase 301 U/L, total bilirubin 485.6 μmol/L, and direct bilirubin 340.2 μmol/L. Abdominal magnetic resonance imaging showed massive lymphomatous lesions filling the peritoneal cavity. Magnetic resonance cholangiopancreatography revealed an external compressive stricture in the superior middle common bile duct and dilation of the intrahepatic and extrahepatic ducts. ERCP was performed unsuccessfully, due to the stricture at the descending junction of the duodenal bulb caused by lymphoma infiltration. So, EUS-guided anterograde common bile duct stenting *via* the stomach was performed.

**Results:**

The patient's bilirubin level decreased significantly in the postoperative period, and no adverse reaction was observed. Computed tomography showed marked shrinking of the abdominal mass after targeted therapy.

**Conclusions:**

Our report suggests that early relief of biliary obstruction may be more beneficial to subsequent chemotherapy when symptoms of lymphoma-associated jaundice are persistently aggravating. Endoscopic ultrasound-guided biliary drainage is a safe, effective and timely alternative approach to treat biliary obstruction when ERCP fails, especially in cases of malignancy caused by extrahepatic bile duct space-occupying lesions.

## Introduction

Endoscopic retrograde cholangiopancreatography (ERCP) is the preferred treatment method for biliary drainage of obstructive jaundice, with a success rate exceeding 90% (1). However, ERCP cannot be applied to about 3%–5% of patients because of the presence of duodenal papillary lesions or periampullary diverticula, history of digestive tract reconstruction, or presence of anatomical abnormalities or malignant biliary obstruction (2). Percutaneous transhepatic biliary drainage (PTBD) and surgery are the traditional alternatives (3, 4). However, PTBD can affect digestive function due to bile drainage, thus both PTBD and surgery are associated with considerable morbidity and mortality (5, 6). Interventional endoscopic ultrasonography (EUS) is a novel approach for drainage of the bile and pancreatic ducts. Endoscopic ultrasonography–guided biliary drainage (EUS-BD) has been recommended as an alternative approach when ERCP fails. Non-Hodgkin lymphoma (NHL) is a rare cause of biliary obstruction (7). Obstructive jaundice can accompany the initial presentation of lymphoma or develop later in the course of the disease (8). Biliary stricture and obstructive jaundice are considered a sign of poor prognosis in lymphoma patients (7). However, little information is available to guide clinicians on the optimal approach to managing obstructive jaundice in lymphoma patients. There are no standard guidelines in the literature, which creates dilemma in management of these patients. It remains unclear whether chemotherapy should precede the biliary drainage procedures in patients with NHL presenting with jaundice. Lymphoma-associated obstruction is generally managed with open surgery, ERCP, or PTBD. In this technical note, we reported on the use of transgastric EUS-BD for the treatment of obstructive jaundice associated with NHL.

## Materials and methods

### Case presentation

**Figure 1:**
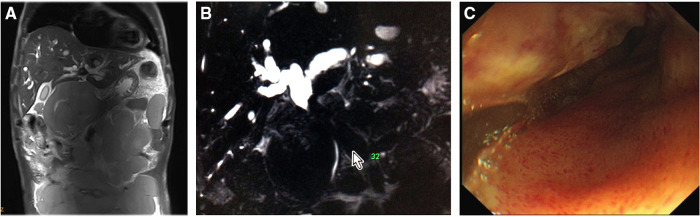
Initial findings. (**A**) Abdominal magnetic resonance imaging showed massive lymphomatous lesions filling the peritoneal cavity. (**B**) Magnetic resonance cholangiopancreatography revealed an external compressive stricture in the superior middle common bile duct and dilation of the intrahepatic and extrahepatic ducts. (**C**) Endoscopic retrograde cholangiopancreatography showed ductal descending-junction stenosis of the duodenal bulb caused by lymphoma infiltration.

A 58-year-old male with no significant personal or familial medical history was admitted with abdominal pain and distension. A submaxillary lymph-node biopsy led to the diagnosis of follicular NHL (grade I–II, phase IV). He reported having dark (tea color) urine and yellowish discoloration of the facial skin and sclera that gradually spread to the whole body. He also complained of persistent abdominal distension, abdominal pain, fatigue, and decreased appetite. Physical examination showed that he had a chronic disease appearance with jaundice of the skin and sclera, and soybean-sized lymph nodes were palpable in the left submandibular region. These enlarged lymph nodes were hard, without significant tenderness, and had poor mobility. Abdominal examination revealed distended abdomen. Multiple masses of different sizes can be touched in the entire abdomen, including the left lower abdomen, with a palpable 15 cm * 20 cm mass with clear boundaries, hard quality, and mild tenderness. Abnormal hepatic function was detected: the patient's alanine aminotransferase (ALT) level was 330 U/L, aspartate aminotransferase (AST) level was 353 U/L, total bilirubin (TBIL) level was 40.33 μmol/L, and direct bilirubin (DBIL) level was 24.21 μmol/L. The patient was treated with medication to protect the liver and decrease the enzyme levels and jaundice, but the jaundice continued to worsen; a hepatic function test performed revealed an ALT level of 168 U/L, AST level of 323 U/L, and TBIL level of 361 μmol/L. Magnetic resonance imaging and computed tomography of the abdomen both revealed numerous filling lymphomatous lesions in the abdominal cavity (Figure 1A), and magnetic resonance cholangiopancreatography showed external pressure stenosis in the middle segment of the common bile duct, expansion of the intrahepatic bile duct, and beak-like expansion of the intrahepatic bile duct (Figure 1B).

**Figure 2:**
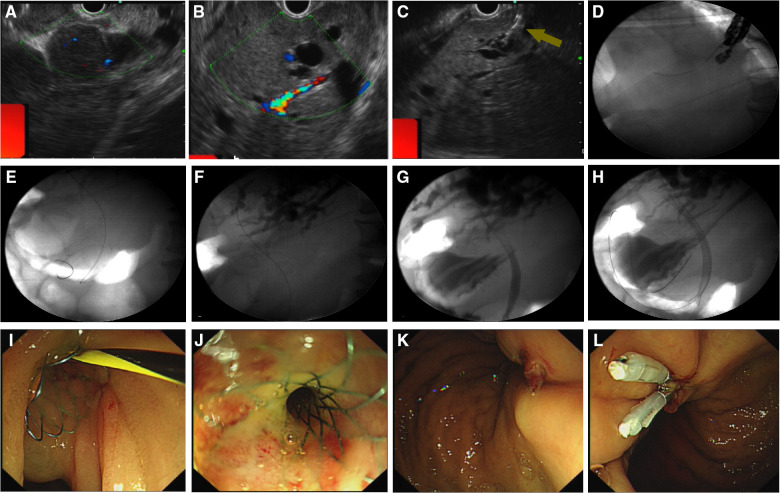
Endoscopic ultrasonography–guided biliary drainage. (**A**) Linear-array ultrasonography showed numerous filling lymphomatous nodules in the abdominal cavity. (**B,C**) The left hepatic duct was located for puncture. (**D,E**) The guidewire was passed through the confluence of the left and right hepatic ducts, and then through the site of common bile duct stricture. (**F,G**) Radiography indicated that the stricture site at the upper common bile duct was 4 cm away from the hepatic portal. (**H**) A bare metal stent (8 cm × 10 mm) was placed transgastrically in the common bile duct in an antegrade manner. (**I,J**) Gastroscopy demonstrated that the stent was passed through and stretched exactly out of duodenal papilla. (**K,L**) The puncture point in the stomach was closed with a titanium clip.

With continued worsening, relief of the obstructive jaundice by puncture drainage was urgently needed to ensure the success of subsequent chemotherapy. ERCP was performed, but failed because of the infiltration of lymphoma into the duodenal bulb, which resulted in narrowing of the descending junction (Figure 1C). Percutaneous transhepatic cholangio-drainage was not performed due to the patient's poor coagulation and the expansion of the right hepatic duct. The patient's jaundice continued to worsen; a hepatic function test performed revealed an ALT level of 211 U/L, AST level of 301 U/L, TBIL level of 485.6 μmol/L, and DBIL level of 340.2 μmol/L. Therefore, EUS-BD was selected as a workable drainage method, and the patient provided informed consent to undergo the procedure. So, EUS-guided transgastric anterograde common bile duct stenting was performed under general anesthesia (Figure 2, Supplementary Video S1). The duration of this operation was about half an hour.

### EUS-BD procedure

First, using a curved linear array echoendoscope (EU-ME2, GF-UCT260; Olympus Medical Systems Co., Tokyo, Japan), we found numerous filling lymphomatous nodules in the abdominal cavity, the largest of which were about 8 cm in diameter (Figure 2A). Next, dilation of the left intrahepatic bile duct (diameter 1.8 cm) was confirmed by linear array ultrasonography. After excluding the presence of regional vasculature by using color Doppler US, the dilated left intrahepatic bile duct was punctured under EUS guidance with a 19G fine-needle aspiration (EchoTip Ultra; Cook Medical, Winston-Salem, NC, USA) (Figures 2B,C). After confirming that the bile duct was correctly punctured on cholangiography, a 0.035- inch guide wire (Jagwire; Boston Scientific, Natick, MA, USA) was introduced into the bile duct. The echoendoscope and needle were angled to facilitate antegrade guidewire passage through the site of obstruction. Then the guidewire was advanced through the confluence of the left and right hepatic ducts and then through the site of common bile duct stricture, becoming tortuous in the descending portion of the duodenum (Figures 2D,E). Subsequently, a needle-knife (Cook Ireland Ltd., Limerick, Ireland) was used to incise the stomach wall along the guidewire and enter the bile duct. A contrast medium was then injected into the bile duct for cholangiography and visualization of the biliary tract; this procedure revealed that the stricture site at the upper common bile duct was 4 cm away from the hepatic portal (Figures 2F,G). Then, a bare metal stent (8 cm × 10 mm) was then placed transgastrically in the common bile duct in an antegrade manner. The contrast medium showed that the upper end of the stent was located at the confluence of the left and right hepatic ducts, and the lower end was located at the opening of the duodenal papilla (Figure 2H). Thereafter, the inferior edge of the stent was checked with a gastroscope. Gastroscopy demonstrated that the stent was passed through and stretched exactly out of the duodenal papilla (Figures 2I,J), so the biliary drainage was performed successfully. Finally, the puncture point in the stomach was closed with a titanium clip (Figures 2K,L, Supplementary Video S1).

## Results

**Figure 3:**
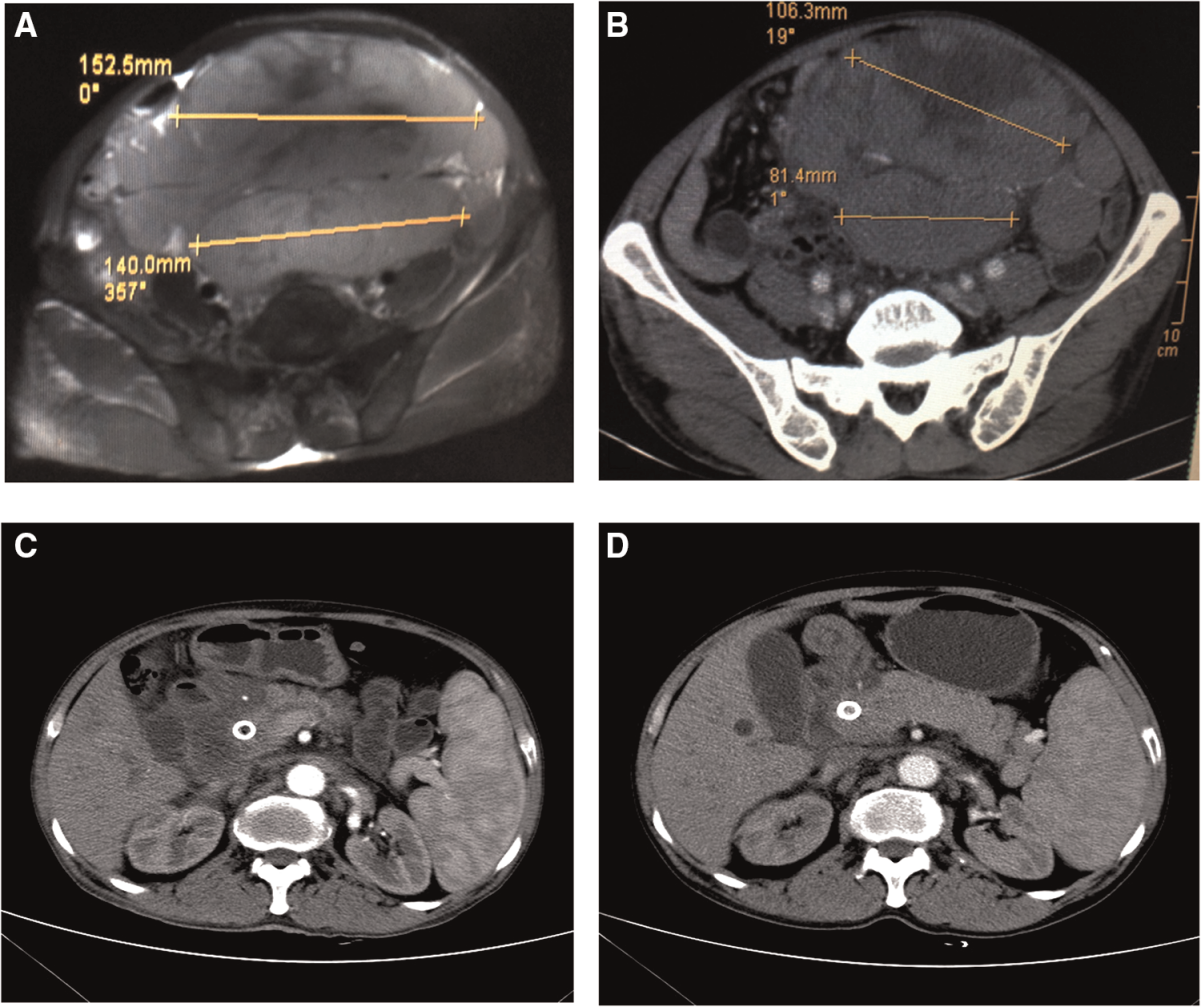
Computed tomography (CT) findings before and after endoscopic ultrasonography–guided biliary drainage. (**A**) A CT image obtained shows a large lump in the abdominal cavity. (**B**) An image obtained (one month after EUS-BD) shows significant shrinking of the abdominal mass. (**C**) CT re-examination (four month after EUS-BD) showed that most of the mass shadows in the abdomen, pelvic cavity, and retroperitoneal cavity were significantly smaller than previously, the pressure on the abdominal viscera was reduced, and the expansion of the portal vein trunk and left and right branches was lessening, with no apparent change in the common bile duct. (**D**) A review total abdominal CT (one year later) showed that abdominal and retroperitoneal lymph node enlargement, little change in internal and external bile duct dilatation,more absorption of gas in the biliary system than previously, and common bile duct stent implantation.

Postoperatively, the patient's jaundice decreased significantly, the liver function test revealed an ALT level of 147 U/L, AST level of 115 U/L, TBIL level of 317.9 μmol/L, and DBIL level of 217.3 μmol/L. No hemorrhage, infection, or sign of pneumoperitoneum appeared. The patient received targeted treatment and chemotherapy [R-CHOP (rituximab, vincristine, pirarubicin, cyclophosphamide, and dexamethasone) and NAP (vinorelbine, cisplatin, and cytarabine)]. One month after EUS-BD, a recheck of liver function revealed an ALT level of 41 U/L, AST level of 27 U/L, TBIL level of 167.1 μmol/L, and DBIL level of 108.2 μmol/L; comparison of CT images showed that the abdominal mass was significantly smaller (Figure 3B) than before (Figure 3A). CT re-examination (four months after EUS-BD) showed further reduction of most mass shadows in the abdomen, pelvic cavity, and retroperitoneal cavity (Figure 3C). After that, the patient was repeatedly admitted to the hospital for chemotherapy, and repeated reviews showed the gradual improvement of his liver function. A total abdominal CT review (one year later) showed that abdominal and retroperitoneal lymph node enlargement, little change in internal and external bile duct dilatation (Figure 3D).

## Discussion

To the best of our knowledge, this is the first study to apply EUS-guided anterograde common bile duct stenting *via* the stomach for obstructive jaundice associated with NHL. The patient's bilirubin level decreased significantly in the postoperative period, and no adverse reaction was observed. Computed tomography showed marked shrinking of the abdominal mass after targeted therapy.

In the case presented here, ERCP failed in a patient with late-stage NHL, lymphomatous filling of the abdominal cavity, and severe obstructive jaundice caused by biliary external pressure stenosis. Preoperative MRCP indicated that external compression stenosis of both upper middle segment of common bile duct and common hepatic duct. Accordingly, we focused on the dilated left hepatic duct, and punctured the intrahepatic bile duct transgastrically, followed by antegrade radiography under EUS guidance and the placement of metal scaffolds in the common bile duct for drainage. This procedure restored the normal physiological flow of bile, thereby saving valuable time for treatment of the primary disease and alleviating further damage to liver function by cholestasis. Compared with other EUS-BD techniques, the strengths of this operation was that it does not require construction of a drainage channel *via*, e.g., hepatic and gastric anastomosis or cholangioenterostomy. Thus, it avoids long-term infiltration of bile to the gastrointestinal mucosa, and greatly reduces the risk of complications such as bile leakage, bile loss, and stent displacement, which was well acceptable and tolerated by patients. Additionally, operators were required to have a high level of guidewire operation to go through FNA puncture needle, bile duct, the obstruction site, the major duodenal papilla and finally to duodenum. The most challenging thing is how to pass through the obstruction site, which should be guided by catheter.

Compared with the plastic stent, the metal stent has a large diameter and is hard to block. It can be expanded rapidly and expand the diameter of the drainage tube simultaneously after implantation. It is able to drain bile into the digestive tract smoothly, and the risk of re-infarction is relatively low. Hong et al. reported that metal stents were associated with significantly longer stent patency, lower reintervention rate and longer patient survival in the palliation of malignant biliary obstruction when compared to plastic stents (9). Some lymphoma patients who require repeated biliary interventions for obstructive jaundice may benefit from early metal stent placement (10). To date, self-expandable metallic stent (SEMS) is widely applied (11). According to whether coated with chemicals designed to prevent tumour ingrowth, SEMS can be divided into covered SEMS and uncovered SEMS. Uncovered SEMS can be better fixed in the lumen due to the mesh embedded into bile duct wall, which is less prone to stent displacement than covered ones. In this case, because of non-intracavitary primary lesion, uncovered SEMS was chosen to achieve a long-term drainage effect. Although covered SEMS is vulnerable to stent displacement, the mechanical properties of the stent can be strengthened to reduce stent displacement (12). In addition, considering the length and shape of the bile duct, a longer stent could be used for better drainage. It is worth noting that a rapid balloon dilation may cause duodenal edema which could lead to bile leakage into abdominal cavity. Hence, nasobiliary drainage could be crucial.

Among all patients with malignant biliary obstruction, NHL accounts for 1%–2% of all cases. Their presentation with obstructive jaundice is mostly secondary to compression of the extrahepatic bile ducts by periportal, perihepatic, or peripancreatic lymphadenopathy, associated tumor lysis, or direct hepatic involvement (13). Although lymphatic masses can compress any part of the biliary tract, the bile duct in the porta hepatis and distal common bile duct are most commonly affected (13, 14). Lymphomatous the abdominal cavity patient was filled with Lymphomatous and it was indicated that ductal stricture under external pressure by MRCP. During the course of chemotherapy, the jaundice was changed to the worse so that alleviation of bile obstruction was imperative. The sequencing of chemotherapy and biliary obstruction removal presents a clinical problem in the treatment of lymphoma-associated obstructive jaundice. Management of these patients is controversia. Many patients with obstructive jaundice have received biliary decompression with external-internal drain, stent placement, or laparotomy (14). These treatments provide relief of obstruction and symptoms, but they do not cure underlying disease; on the other hand they could delay definitive treatment administration. Dudgeon and Brower argued that additional biliary decompression is unnecessary (15), and that combined chemotherapy is the best initial treatment, as it can rapidly relieve biliary obstruction. However, most chemotherapeutic drugs, including anthracycline, must be metabolized by the liver, and cholestasis further aggravates their hepatic toxicity. The data of Ross and Egwim are more in favor of early biliary decompression. Their study shows that biliary decompression can be effectively achieved with ERCP or PBD, thus avoiding surgery with its associated morbidity (7) and the potential for heightened risk of hepatic toxicity from chemotherapy. The early removal of biliary obstruction can reduce the risks posed by subsequent chemotherapy, extending patients' survival time (10).

Lymphomatous obstructive jaundice is mainly relieved by surgery, ERCP or PTBD. Endoscopic ultrasound-guided puncture and drainage were used as a more uninterrupted and achievable way. This method retained the bile natural drainage path, by which liver function recovered without any postoperative complication such as bleeding, pneumoperitoneum, infection, etc. Thereby, it was acceptable for patients. There is increasing evidence to suggest that EUS-guided biliary drainage (EUS-BD) is a safe and effective treatment alternative for patients with malignant biliary obstructions after failed endoscopic retrograde cholangiopancreatography. Itonaga et al. showed that EUS-guided choledochoduodenostomy (EUS-CDS) is superior to ERCP in terms of stent patency and safety for the first-line drainage of malignant distal biliary obstruction (16). Chin et al. found that EUS-BD are effective EUS-based techniques for managing patients with malignant biliary obstructions following ERCP failure (17). Our report shows the application of EUS-guided anterograde common bile duct stenting *via* the stomach for obstructive jaundice associated with NHL. When chemotherapy cannot improve the symptoms of lymphoma-related jaundice, the early use of EUS technology to relieve biliary obstruction will be more beneficial to the patient's subsequent treatment. However, larger randomized studies are needed to compare different drainage methods to produce more robust evidence and guide practice. Further follow-up and more studies are required to further elucidate the most appropriate treatment modality.

However, the described technique has some limitations. There is no standardized approach in terms of the technical aspects. There are limited devices dedicated to performing EUS-BD. Major limitations also include the increased risk of complications. Where repeat cannulation attempts or alternate cannulation methods can be performed when the initial attempt fails, repeat attempts at EUS-BD in a single setting may result in a higher risk of severe complications such as bile leak, infection, and bleeding (18). Hence, these procedures should be performed by expert endoscopists in high-volume centers.

In conclusion, we described a transgastric endoscopic ultrasonography-guided bile drainage technique for the treatment of obstructive Jaundice induced by Non-Hodgkin Lymphoma. Our report suggests that early relief of biliary obstruction may be more beneficial to subsequent chemotherapy when symptoms of lymphoma-associated jaundice are persistently aggravating. Endoscopic ultrasound-guided biliary drainage is a safe, effective and timely alternative approach to treat biliary obstruction when ERCP fails, especially in cases of malignancy caused by extrahepatic bile duct space-occupying lesions, such as lymphoma-related obstructive jaundice.

## Data Availability

The original contributions presented in the study are included in the article/**Supplementary Material**, further inquiries can be directed to the corresponding author/s.
